# Mediterranean Diet Adherence and Subjective Well-Being in a Sample of Portuguese Adults

**DOI:** 10.3390/nu12123837

**Published:** 2020-12-16

**Authors:** Vanda Andrade, Rui Jorge, María-Teresa García-Conesa, Elena Philippou, Marika Massaro, Mihail Chervenkov, Teodora Ivanova, Viktorija Maksimova, Katarina Smilkov, Darinka Gjorgieva Ackova, Lence Miloseva, Tatjana Ruskovska, Georgia Eirini Deligiannidou, Christos A. Kontogiorgis, Paula Pinto

**Affiliations:** 1 Polytechnic Institute of Santarém, School of Agriculture, 2001-904 Santarém, Portugal; vanda.andrade@esa.ipsantarem.pt (V.A.); rui.jorge@esa.ipsantarem.pt (R.J.); 2 Life Quality Research Centre (CIEQV), IPSantarém/IPLeiria, 2040-413 Rio Maior, Portugal; 3 Centro de Investigação Interdisciplinar Egas Moniz (CiiEM), Instituto Universitário Egas Moniz, 2829-511 Monte de Caparica, Portugal; 4 Research Group on Quality, Safety and Bioactivity of Plant Foods, Campus de Espinardo, Centro de Edafologia y Biologia Aplicada del Segura-Consejo Superior de Investigaciones Científicas (CEBAS-CSIC), P.O. Box 164, 30100 Murcia, Spain; mtconesa@cebas.csic.es; 5 Department of Life and Health Sciences, School of Sciences and Engineering, University of Nicosia, Nicosia 1700, Cyprus; Philippou.e@unic.ac.cy; 6 Department of Nutritional Sciences, King’s College London, London SE1 9NH, UK; 7 National Research Council (CNR), Institute of Clinical Physiology, 73100 Lecce, Italy; marika@ifc.cnr.it; 8 Slow Food in Bulgaria, 9 Pierre De Geytre St. bl. 3, 1113 Sofia, Bulgaria; vdmchervenkov@abv.bg; 9 Faculty of Veterinary Medicine, University of Forestry, 1797 Sofia, Bulgaria; tai@bio.bas.bg; 10 Department of Plant and Fungal Diversity and Resources, Institute of Biodiversity and Ecosystem Research, Bulgarian Academy of Sciences, 1113 Sofia, Bulgaria; 11 Faculty of Medical Sciences, University Goce Delcev, str. Krste Misirkov, No. 10-A, POB 201, 2000 Stip, Republic of North Macedonia; viktorija.maksimova@ugd.edu.mk (V.M.); katarina.smilkov@ugd.edu.mk (K.S.); darinka.gorgieva@ugd.edu.mk (D.G.A); lence.miloseva@ugd.edu.mk (L.M.); tatjana.ruskovska@ugd.edu.mk (T.R.); 12 Laboratory of Hygiene and Environmental Protection, School of Medicine, Democritus University of Thrace, 68100 Dragana, Alexandroupolis, Greece; edeligia@med.duth.gr (G.E.D.); ckontogi@med.duth.gr (C.A.K.); 13 Research Unit of Polytechnic Institute of Santarém, 2001-904 Santarém, Portugal

**Keywords:** Mediterranean diet, MEDAS score, subjective well-being, life satisfaction, Portuguese adults

## Abstract

The Mediterranean diet (MD) and other lifestyle characteristics have been associated with well-being, a broad multiparameter concept that includes individual’s subjective assessment of their own well-being (SWB). Some studies have suggested that diet influences SWB, thus, this work aimed to add novel information on the association of MD and SWB in a sample of Portuguese adults. Data on sociodemographic, economic, lifestyle, diet, and SWB were collected through a self-filled online questionnaire. MD adherence was assessed by the Mediterranean Diet Adherence Screener (MEDAS) score. Results showed a moderate adherence to the MD in 490 Portuguese adults (mean MEDAS of 7.4 ± 2.1). A higher MD adherence was found to be significantly positively associated with women, employed individuals, a higher number of meals per day, and those with frequent contact with nature (*p*-value < 0.0025, using Bonferroni adjustment). As a novelty, this study divided the participants into low SWB, medium SWB, and medium to high SWB profiles (3.9 ± 1.0; 6.2 ± 1.0; 8.2 ± 1.3, respectively; *p*-value < 0.05), which reported significantly increasing MEDAS scores (6.5 ± 2.1; 7.3 ± 2.1; 7.8 ± 1.9; respectively, *p*-value < 0.05).

## 1. Introduction

The Mediterranean diet (MD) represents the heritage of millennia of exchanges among people, cultures and foodstuffs around the Mediterranean basin [1]. The traditional MD has emerged as a healthy dietary pattern since the Seven Country Study showed that the dietary pattern common to olive growing areas of the Mediterranean region was associated to lower rates of all-cause and coronary heart disease death [2]. This dietary pattern is characterized by a high consumption of low processed plant foods (cereals, fruits, vegetables, legumes, tree nuts, seeds and olives), olive oil as the main source of dietary lipids, moderate intake of fish and seafood (depending on the proximity to the sea), moderate wine, and low intake of eggs, meat, dairy, and animal fat [1]. After the recognition of the MD as an Intangible Cultural Heritage of Humanity by UNESCO in 2010, a Mediterranean diet pyramid was defined, reflecting the concept of MD as a way of life within the Mediterranean societies [3]. This pyramid established dietary daily, weekly and occasional food consumption guidelines, and included characteristics such as consumption of seasonal products and eating the main meals convivially, with family or friends [3]. Lifestyle factors such as regular practice of moderate physical activity and adequate night sleep were also part of a healthy and balanced lifestyle [3]. Overall, MD and other lifestyle characteristics have been associated with health and well-being [4,5,6].

In the last decade, the concept of well-being has been introduced to health-related disciplines, social sciences and food marketing, with a diversity of meanings and measures [7]. The concept has also been incorporated in the World Health Organization (WHO) definition of health: “health is a state of complete physical, mental and social well-being and not merely the absence or disease or infirmity” [8]. In 2015, the WHO Regional Office for Europe has targeted the well-being of the European population as one of the priorities in the Health 2020 Monitoring Framework [9]. Well-being is a broad concept that integrates objective social and environmental factors, such as availability of social support and sanitation facilities, unemployment rate, income and education, as well as an individual’s subjective assessment of their own well-being, conceptualized as subjective well-being (SWB) [9]. SWB is a psychological construct defined as a normally positive state of mind that involves the whole life experience [10]. As specified in the Organisation for Economic Co-operation and Development (OECD) Guidelines on Measuring Subjective Well-being [11], SWB indicators are self-assessed psychological factors with two complementary sub-dimensions: (i) a hedonic sub-dimension, which includes life evaluation, a cognitive component reflecting overall life satisfaction, and an affective component, related to perceptions of positive and negative affective states (feelings of joy and happiness; feelings of depression and worry), and (ii) an eudemonic sub-dimension, related to meaning and purpose of life.

Some studies have suggested that diet and SWB may be related. For instance, the adherence to the MD has been associated with better overall perceived quality of life [12] and with better health related quality of life, with a stronger association with mental health than with physical health [5]. While exposure to unhealthy dietary habits may be associated with negative mood and predispose to depression, plant food intake seems to be associated with greater vitality and inversely associated to perceived stress and depression [13,14,15,16]. In Portugal, a study with 10,153 Portuguese adults has observed that individuals with a “meat dietary pattern” (high frequency of meat consumption; low frequency of soup, vegetables, fresh fruit, fish and dairy) reported depression symptoms more often than individuals with a “fruit and vegetables dietary pattern” (high frequency of consumption of soup, vegetables, fresh fruit, fish and dairy; low frequency of meat consumption) [17]. Nevertheless, some other studies, observed no relationship between diet and depression rates in healthy older adults [18], or between MD and psychological well-being [15], rendering it difficult to reach firm conclusions. Furthermore, these associations may be influenced by other health behaviours that also impact on well-being, such as physical activity and sleep quantity [19].

In Portugal, data on the adherence to the MD are limited, however, Portugal’s food availability data show that since the 1960s the Portuguese dietary pattern has been diverging from the MD [20,21]. Furthermore, to our knowledge, there are no studies on the association of MD and SWB in Portuguese adults. Thus, the main aims of this study were to assess adherence to MD by a validated instrument (14-point Mediterranean Diet Adherence Screener (MEDAS) [22,23], and investigate the relationship between MD and SWB.

## 2. Materials and Methods

### 2.1. Study Design and Ethics

The study was approved by the Ethic Committee of the Research Unit of Polytechnic Institute of Santarém (Document 022019Agrária), and complies with European Regulation on Data Protection [24]. The study was designed to assess MD adherence with the previously validated 14-point MEDAS [22,23], as well as factors of lifestyle and SWB. A structured questionnaire was constructed in Google Forms for collection of data. Enrollment in the study was performed by the dissemination of the questionnaire through institutional mailing lists, social media and personal contacts of the researchers involved in the study, mostly in the region of Lisbon and Alentejo. Eligible participants to fill the questionnaire were adults (age ≥ 18 years). No gender, educational, health, social, and cultural selection were intended at this phase. The questionnaire was confidential and was filled anonymously online. Collection of anonymous data occurred from May to December 2019. A total of 505 participants completed the questionnaire. Before analysis, fifteen responses were excluded from the analysis due to no consent of data utilization (5), and age below 18 years (1). Two other exclusion criteria were applied before data analysis: nationality different from Portuguese living in Portugal, or Portuguese living abroad (9). Thus, data from 490 Portuguese adults (age ≥ 18 years), living in Portugal were eligible for analysis.

### 2.2. Data Collection

The questionnaire was developed under the frame of an international project involving a consortium of seven countries [22]. The design of the questionnaire was done according to the Organisation for Economic Co-operation and Development (OECD) recommendations [11], and consisted of 57 questions (Supplementary Materials S1). The questionnaire began with an introductory explanation of the study and the nature of the participation, with a question at the end asking for authorization to use the data anonymously for statistical analysis and scientific publication. After consenting to fill the questionnaire, the participant proceeded to the following sections of the questionnaire, which included questions on sociodemographic data, SWB, health status, lifestyle, and food habits. The present work is a sub-study of the complete questionnaire. In this sub-study the following data were used for analysis (questions 2.1 to 2.13; 2.20; 2.23 to 2.30; 2.33; 2.36; 3.1 to 3.15 from the complete questionnaire): (i) sociodemographic data included, age (years), sex, marital status (single, married or living with partner in a stable relationship, divorced, separated, widowed), household size, education (non-graduated, graduated, master degree, PhD), employment status (student, employed, unemployed part of the year, unemployed, retired), and household income; (ii) health and nutritional status information included self-reported diagnosed pathology, weight (kg) and height (m), used to calculate BMI(Body Mass Index, weight (kg)/height^2^ (m^2^); (iii) lifestyle data included smoking (smoker or non-smoker), physical activity (regular or non-regular practice), sleeping hours (less than six hours per night, six to seven hours per night, seven to eight hours per night, eight to ten hours per night, more than ten hours per night), frequency of contact with nature (never or occasionally, sometimes, frequently or almost all the time), and frequency of contact with family and friends (never or occasionally, sometimes, frequently or almost all the time); (iv) SWB data included the five core questions recommended from the OECD [11], assessed with a 10 point Likert-type scale, where 0 = not at all to 10 = completely/all the time): one question about life global evaluation (overall, how satisfied are you with your life as a whole these days?); three affect questions (how happy did you feel during the last week?; how worried did you feel during the last week?; did you feel depressed during the last week?); one eudemonic question (to what extent do you feel that the things you do in life are worthwhile?); (v) the 14 food frequency questions used to calculate the 14-MEDAS [22], and one question on the number of meals per day.

### 2.3. Data Analysis

Statistical analysis was performed with the Statistical Package for the Social Sciences (SPSS) 26 statistical package for Windows (SPSS, Inc., Chicago, IL, USA). The presence of nominal, ordinal and scale variables, as well as testing scale variables for normality and heteroscedasticity, by Kolmorogov–Smirnoff and Levene tests, rendered the use of non-parametric analysis as the best choice [25]. Sociodemographic, lifestyle, BMI, MD and SWB data are represented as % for ordinal or nominal variables, and as median and interquartile range (IQR) for scale variables. To facilitate comparison with other studies, we also present mean and SD for scale variables. Mann–Whitney U tests for ordinal and scale variables, or chi-square tests for nominal variables were used to assess differences between genders (significant for *p*-values < 0.05) [25].

Non-parametric partial correlations of all sociodemographic, BMI, lifestyle, and SWB variables with the 14-MEDAS were performed. This type of correlation allowed to control all the identified potential confounders (sex, age, years of residence in Portugal, education, marital status, employment status, net income, smoking, time spent in nature, meals per day, satisfaction with life as a whole, feeling that things do in life are worthwhile, feelings of happiness, worry and depression). These confounders were identified by the observance of significant bivariate correlations with 14-MEDAS score and, if uncontrolled could bias the results. Additionally, it was considered that when conducting multiple analyses on the same dependent variable (14-MEDAS score), there is an increased chance of committing a type I error. A type I error implies a higher chance for a false positive, or in other words, rejecting the null hypothesis when it should not. For this reason, the *p*-value was adjusted by Bonferroni’s Correction, which is one of several methods used to counteract the problem of multiple comparisons [26]. Data are represented as Spearman’s partial correlation values/*p*-values (significant for *p*-values < 0.0025).

To analyse association of 14-MEDAS with SWB, the five core SWB items were used to group subjects into profiles. Two-Step Clustering analysis is an exploratory tool designed to reveal natural groupings within a dataset that would otherwise not be apparent. Hence, this analysis was performed applying a Log-likelihood distance measure and the Schwarz’s Bayesian clustering criterion, for which each solution is compared against each other [25,27], and was used to determine the best number of groups. This methodology leaded to the creation of three profiles: one including the subjects with high SWB (profile 1), another one with subjects exhibiting intermediate SWB (profile 2) and profile 3 including the subjects with the low SWB. Then for each profile, global 5-item SWB indexes were determined by calculating the average of the five SWB parameters (an inverted scale, 10- “x”, was applied to two variables: feeling of worry and feeling of being depressed). The validity of the clusters and 5-item SWB indexes for each profile were verified by Mann–Whitney U tests (significant differences for *p*-value < 0.05). The SWB parameters of each profile are represented as mean ± SD. The profiles created according to SWB indexes were compared with 14-MEDAS by Mann–Whitney U tests. Differences were considered significant when *p*-value < 0.05. Classes of adherence to MD were defined as low adherence (14-MEDAS ≤ 5), moderate adherence (14-MEDAS between 6 and 9), and high adherence (14-MEDAS ≥ 10) [28].

## 3. Results

### 3.1. Sociodemographic Characteristics of the Participants

The main sociodemographic characteristics of the participants are shown in Table 1. From the 490 individuals that entered this study that were of Portuguese nationality and living in Portugal, more than 50% had been living in Portugal for more than 20 years, were women, and were young people of ages up to 34 years old (Table 1). Most participants were single or engaged in a stable relationship (Table 1), and the median household size was of three (IQR 2) persons (data not shown in Table 1). Significant differences in the distribution between men and women were observed for education level (*p*-value = 0.025), employment status (*p* = 0.003), and net income (*p*-value = 0.024). Secondary level predominated in men, while university level predominated in women (Table 1). Although most of the participants were employed (70%), a higher percentage of students were men, while a higher percentage of unemployed participants was observed in women (Table 1). The monthly net income was referenced to Portuguese Social Support Index (PSSI) in 2020 (438.81 Euros) and half of the respondents had incomes higher or equal to four times the PSSI value, with men reporting higher income values (Table 1).

### 3.2. MD Adherence, Lifestyle Habits and Nutritional Status

Adherence to MD and other lifestyle habits are presented in Table 2. The median adherence to MD was assessed by 14-MEDAS, presenting a median score of 7 (IQR 3) and a mean of 7.4 (SD 2.1) (Table 2). A significant difference in the distribution of men and women among the MEDAS categories was observed, with women showing a higher adherence to MD (*p* value < 0.001, Table 2). Using the criteria defined in the PREDIMED (Prevención con Dieta Mediterránea) trial [28], a low percentage of the study population (17.1%) could be classified as having a high adherence to the Mediterranean diet, with most of the participants falling within a moderate adherence (62.7%), and 20.2% having a low adherence. A significant difference was also observed in the number of meals per day, with a higher percentage of women reporting four or five meals per day, compared to a higher percentage of men reporting eating three or four meals per day (*p*-value < 0.001). Regarding other lifestyle factors, about 80% of the respondents were non-smokers, and slept 6 to 8 h per night; most of the participants did not practice physical activity regularly (Table 2). Significant differences in the distribution between men and women were observed for smoking (26.6% men, 15.8% women, *p*-value = 0.005).

Most participants reported not having a diagnosed pathology, and had a BMI classified as normal weight (Table 3). Men exhibited significantly higher BMI values than women (*p*-value < 0.001), with median values of 24.3 and 22.6 kg/m^2^, respectively (Table 3). Overweight was more prevalent among men, but obesity was higher among women (Table 3).

### 3.3. Subjective Well-Being

Regarding SWB, a median value of 7 was obtained for the five-item SWB index, and no significant differences in the distribution were observed between men and women (Table 4). Globally, the highest score was observed for the eudemonic question (feeling that life is worthwhile), followed by the satisfaction with life and happiness (Table 4). Scores of feeling depressed were the lowest and showed a larger interquartile range (Table 4).

Cluster analysis lead to grouping of participants into three different SWB profiles, ranked by the five-item SWB index: profile one, 8.2 ± 1.3; profile two, 6.2 ± 1.6; profile three, 3.9 ± 1.0 (Table 5). More than half of the participants fitted into profile two, with profile three showing the lower percentage of participants (Table 5).

### 3.4. Variables Influence on the MD Adherence: Exploring the Relationship between MD and SWB

Sociodemographic, BMI, lifestyle and SWB parameters were tested for correlations with 14-MEDAS score (Table 6). Despite the fact that no strong associations were found (ρ values between 0.1 and 0.3 for all the studies factors), weak significant positive correlations with 14-MEDAS were observed for sex (*p*-value = 0.002), employment status (*p*-value = 0.002), time spent in nature (*p*-value < 0.001) and number of meals per day (*p*-value < 0.001) (Table 6).

**Figure 1:**
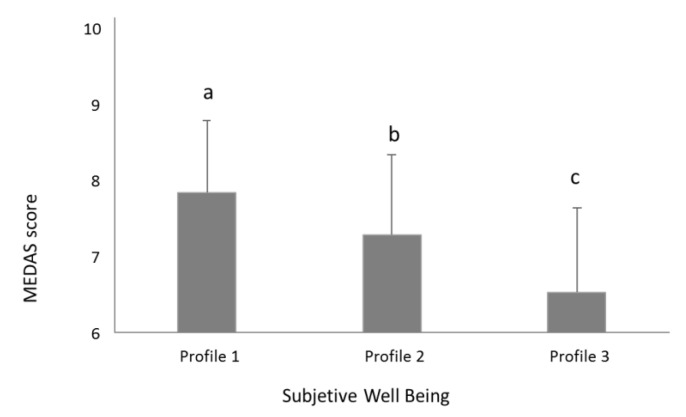
Adherence to the Mediterranean diet of subjects clustered in Profiles 1, 2 or 3 according to their Subjective well-being (SWB): different letters indicate significant differences between profiles (*p* < 0.05). MEDAS (Mediterranean Diet adherence screener)

As shown in Table 6, when the five items of SWB were tested individually for correlations with MEDAS score, no significant correlations were observed for any of the SWB parameters, when considering Bonferroni corrections (*p*-value < 0.0025). However, as shown in Figure 1, the MEDAS score is lower in the profile with the lower five-item SWB index (profile three, MEDAS = 6.5 ± 2.1) compared with the other two profiles (profile two, MEDAS = 7.3 ± 2.1; profile one, MEDAS = 7.8 ± 1.9; *p* < 0.001 for profile one and *p* = 0.022 for profile two) (Figure 1). In the same way MEDAS score in subjects from profile two was significantly lower (*p* = 0.005) than the one found in profile one subjects (Figure 1).

## 4. Discussion

The scarcity of data on the adherence to the MD of Portuguese adults, as well as on the assessment of SWB enhances the relevance of this study. MD adherence of Portuguese adults was assessed with 14-MEDAS, in a sample of 490 individuals, showing a mean 14-MEDAS score of 7.4 ± 2.1. Two other studies on Portuguese convenience population samples in the same region as this study reported similar MEDAS scores: 7.3 ± 2.2, with participants mostly from primary care centers and a Medical School (*n* = 224) [30], and 6.8 ± 2.3 with participants from a Portuguese university (*n* = 305) [31]. With regard to adherence to the MD, the results from this study show a low percentage (17.1%) classified as high adherence to the MD (14-MEDAS score of 10), still slightly higher than another study undertaken with 5653 Portuguese adults covering all the territories of Portugal, where 12% of the participants scored a 14-MEDAS value of 10 and 88% had a 14-MEDAS score below 10 [32]. Most of the participants in the present study could be classified in the range of moderate MD adherence (62.7% with 14-MEDAS scores between six and nine). Results from this study highlight significant differences in MD between genders, where women scored higher 14-MEDAS values than men (6.6 ± 2.2 for men and 7.7 ± 2.0 for women, *p*-values < 0.001). Gender differences with regard to healthy dietary patterns have been observed in other Portuguese studies. A representative national study showed that women are more likely to adopt a healthier dietary pattern, characterized by high intake of fruit and vegetables and a low intake of meat, while men tend to adopt a dietary pattern with high consumption of meat and low intake of fruits and vegetables [17]. Regarding adherence to MD, in a study with university students, women had a statistically significant higher 14-MEDAS score than men [31].

In other European Mediterranean countries, the adherence to the Mediterranean dietary pattern assessed by 14-MEDAS varies. For instance, in Spain, the PREDIMED study [28], which enrolled 7447 asymptomatic participants at high risk for coronary heart disease aged 55–80 years old, showed a MEDAS score of 8.6 ± 2.0, and in a sample of Spanish university students (*n* = 310) the mean 14-MEDAS score was 7.0 ± 2.0 [33]. On the other hand, in Greece a non-probability sample of 236 students, aged 19–30 years old showed a mean 14-MEDAS score of 6.4 ± 1.9 [34], with the 14-MEDAS scores for women being statistically significant higher compared to their male counterparts. Outside the Mediterranean region, the mean 14-MEDAS scores tend to be lower than in Mediterranean countries both in European and in non-European countries. For instance, in the United Kingdom, in a study performed in Bristol in 96 adults with a high cardiovascular risk, the mean 14-MEDAS score was 5.5 ± 2.1 [35], and in South Korea, in a study run at a health check-up centre with 211 participants, the 14-MEDAS score was found to be 6.2 ± 2.2 [36]. Naturally, being located further from the Mediterranean region, the cultural background, food habits and foods availability lowers the chances of higher adherence to the Mediterranean dietary pattern. Nevertheless, since the MD has consistently shown to promote health, both in observational studies and randomized trials [5], its assessment in different countries and in different population samples is of the utmost importance.

After adjustment for sociodemographic, socioeconomic and lifestyle factors, this study showed a significant correlation (*p*-value < 0.002) of MD adherence with employment status and gender, with employed individuals and women showing higher levels of adherence than unemployed individuals and men. However, it is important to point that these were weak correlations (correlation coefficients between 0.1 and 0.2). Although Bonferroni correction did not render net income as statistically significant (*p*-value > 0.003), there was a tendency of higher incomes being associated with higher 14-MEDAS scores than lower incomes. Nevertheless, a careful interpretation of the results is needed, since 17.5% of the participants did not report the net income, which might be associated with lower levels of income. Other studies have reported healthier dietary patterns or higher adherence to MD in women and individuals with higher income [17,37,38,39]. Most of these studies also found a positive association of MD adherence to other sociodemographic factors such as older age and being married or living as a couple [18,38,40], and other socioeconomic factors such as a higher education level [37,40]. However, no general rule can be drawn; some studies have found no association of MD adherence with age and gender [40], and one study has observed different results for different instruments measuring MD adherence in the same population [37], suggesting that associations between healthy eating and gender or age depend on the assessment instrument. The same may also be true for lifestyle factors. No statistically significant correlates were found in this study between the adherence to the MD assessed by MEDAS and physical activity, or smoking, despite other studies reporting a positive association between the adherence to the MD, assessed by methods other than 14-MEDAS, with physical activity [40,41], and an inverse association with smoking, and sedentary behaviours [37]. In this study, a lifestyle behaviour that showed a weak significant correlation with MD adherence was the number of meals (*p*-value < 0.001). This is partially in line with another Portuguese study that observed a higher number of meals in participants following a diet rich in fruit and vegetables [18]. The present study also showed, for the first time, that a higher frequency of contact with nature may be related to a higher adherence to the MD (*p*-value < 0.001). The latter should be cross evidenced further with larger samples that include not only urban populations but also participants from the countryside, where stress levels and skewed demography could present different correlations. In fact, it has been shown that the relationship between sociodemographic and economic factors, and dietary intake patterns differ in rural versus urban areas [42].

Although no causal relationship has yet been demonstrated, several studies have been accumulating evidence on the positive association between healthy food habits, particularly consumption of vegetables and fruits, and quality of life, happiness, and feelings of depression, [43,44]. Thus, it was expected that MD would have the same beneficial association. In fact, some studies have shown a positive association between MD and quality of life in healthy adults [5,45], and an inverse association with depression risk, with significant dose–response relationships [17,46]. On the other hand, enhanced SWB has been linked to a better health status, and healthy behaviors [47,48]. Most of the performed studies were observational and focused on the hedonic dimension of well-being, showing that higher levels of life satisfaction and positive affect were correlated with a lower incidence of cardiovascular disease, diabetes, and cancer [49]. Other studies have also looked for associations between the hedonic sub-dimension and diet. For example, in the SUN study, the authors assessed quality of life with the Spanish version of the quality of life questionnaire (QoL), which includes questions on depression and happiness in the emotional well-being dimension. The highest significant correlation for emotional well-being was fruit consumption [45], which is a type of food highly consumed in MD. Furthermore, a study with people above 60 years showed a direct relationship between the adherence to the MD and life satisfaction of women but not for men [50]. Some recent research has shown that the eudemonic dimension is also associated with a reduced risk of chronic diseases and higher incidence of healthy behaviours, such as physical activity and fruit and vegetables intake [51,52].

To our knowledge, this is the first study where a higher adherence to MD was observed in adult participants with higher SWB, as a concept including both eudemonic and hedonic well-being. It has been suggested that both hedonic and eudemonic constructs should be included to best capture associations of psychological well-being and longevity [49], and this study applied this concept to study associations between SWB and MD. Eudemonic SWB assessed the extent that the individual considered that life was meaningful and worthwhile. Hedonic SWB assessed overall life satisfaction, and feelings of happiness, depression, and worry. No significant differences in the distributions between men and women in any of the five SWB components were observed, in line with results reported by the statistical office of the European Union (EUROSTAT), regarding overall life satisfaction [53]. The five components were combined to create three SWB profiles, with the average of the five components defining a five-item SWB index (8.2 ± 1.3, 6.2 ± 1.0, 3.9 ± 1.0 for profiles one, two, and three, respectively). Classifying the five-item SWB index, according to the EUROSTAT classification of overall life satisfaction (0 to 5, low; 6 to 8, medium, 9–10, high) [53], profile one would be classified as medium to high SWB, profile two as medium SWB, and profile three as low SWB. Although all profiles could be classified as moderate adherence to MD (14-MEDAS score between six and nine), the present study has shown that MD adherence significantly increases (*p*-value < 0.01) from low SWB (profile three, mean 14-MEDAS score of 6.5) to medium/high SWB (profile one, mean MEDAS score of 7.8).

It is interesting to note that the mean of overall life satisfaction (hedonic sub-dimension) of profiles one (8.4 ± 1.0), and two (7.2 ± 1.0) is higher than the reported mean values for the Portuguese population in 2018 (6.7 [53]), and the mean value observed in profile three is much lower (4.7 ± 1.6). The same is true for the eudemonic sub-dimension, with a mean value of 8.9 ± 0.9, 7.7 ± 1.0, and 5.1 ± 1.7, for profiles one, two, and three, respectively, compared to the average value reported in 2013 for the Portuguese population (7.5 [53]).

Some limitations should be considered when interpreting the results: the analysis was conducted on a relatively low number of individuals, and the study design only allowed to define associations, not causal relations. Additionally, the use of self-filled frequency questionnaires to assess dietary patterns, may introduce an overestimation of foods considered healthy and underestimation of foods considered non-healthy. Furthermore, the interpretation of frequencies such as occasionally, sometimes, and frequently, may be different among participants, when assessing frequency of contact with nature, or family and friends. It must also be noted that 17.5% of the respondents did not answer the question on net income, which may have conditioned the analysis of correlation between net income and MD adherence. On the other hand, regarding SWB self-assessment, the fact that individuals are in their natural settings when they complete the questionnaire, may prevent distortion of results due to unusual circumstances. Another strength of the study is the eligibility of participants being restricted to Portuguese citizens living in Portugal, and excluding citizens from other nationalities living in Portugal, or Portuguese citizens living abroad, which prevents cultural and linguistic factors that may introduce biases in country-level MD adherence and SWB.

## 5. Conclusions

In conclusion, this study shows a moderate adherence to the Mediterranean dietary pattern in a study sample of 490 Portuguese adults. Although the level of correlation was weak, a higher level of adherence was found to be positively associated with women, employed individuals, a higher number of meals per day, and those with frequent contact with nature. As a novelty, this study assessed both hedonic and eudemonic SWB, showing that low SWB, medium SWB, and medium to high SWB profiles reported significantly increasing MEDAS scores.

The assessment of SWB is becoming increasingly important as mental illness escalates in a society subjected to many kinds of economic, social, and environmental stress. Furthermore, general health and mental wellness are intimately linked to healthy behaviors, with MD playing a central role as a multicomponent concept including healthy, sustainable food habits, and other lifestyle and social factors that impact on health and society. Thus, further exploring MD and SWB in larger samples will uncover co-benefits of this relationship, so that impactful policies may be implemented to enhance society well-being as a whole.

## Figures and Tables

**Table 1:** Participants’ sociodemographic characteristics and gender comparison.

	Total	Men	Women	*p*-Value ^(1)^
Gender (%/N)		28.5/139	71.5/349	
Age (median (IQR))	34.0 (22.0)	29.0 (27.0)	36.0 (22.0)	0.34
(mean ± SD)	36.5 ± 13.6	36.0 ± 15.3	36.6 ± 12.8	
Age categories (%/N)				
<35 years	50.8/249	57.6/80	48.1/168	
35–54 years	35.5/174	21.6/30	41.3/144	
≥55 years	13.7/67	20.9/29	10.6/37	
Years in Portugal (%/N)				0.417
<10 years	15.1/73	13.7/19	15.4/53	
10 to 20 years	18.8/91	17.3/24	19.5/67	
>20 years	66.2/321	69.1/96	65.1/224	
Marital status (%/N)				0.115
Single	46.9/230	53.2/74	44.4/155	
Married or analogous relationship	45.1/221	38.8/54	47.6/166	
Divorced or separated	7.1/35	7.2/10	7.2/25	
Widowed	0.8/4	0.7/1	0.9/3	
Education level (%/N)				**0.025**
Middle school	3.5/17	5.0/7	2.9/10	
High school	26.5/130	37.4/52	22.3/78	
University	42.9/210	30.2/42	47.9/167	
Master	20.8/102	20.9/29	20.9/73	
Ph.D.	6.3/31	6.5/9	6.0/21	
Employment status (%/N)				**0.003**
Student	21.6/106	30.9/43	17.8/62	
Employed	70.0/343	62.6/87	73.2/255	
Unemployed part of the year	2.9/14	1.4/2	3.4/12	
Unemployed or Housework	2.5/11	1.4/2	2.6/9	
Pensioner (retired, disability)	2.7/13	3.6/5	2.3/8	
Monthly net income (euros) (%/N)				**0.024**
<PSSI ^(2)^	1.5/6	0.0/0	2.1/6	
≥PSSI to 2× PSSI	10.4/42	6.9/8	11.9/34	
≥2× PSSI to 4× PSSI	37.6/152	35.3/41	38.8/111	
≥4× PSSI	50.5/204	57.8/67	47.2/135	

Sample size is not constant due to missing data in different variables (sex, *N* = 488; age, marital status, education level, *N* = 490; employment status, *N* = 487; monthly income *N* = 404). IQR= interquartile range; SD= standard deviation ^(1)^ Mann–Whitney U tests were used to assess gender differences in ordinal and scale variables; chi square tests were used for nominal variables (significant differences in bold when *p*-values < 0.05); ^(2)^ PSSI: Portuguese Social Support Index in 2020. (438.81 €).

**Table 2:** Participants’ MD adherence, other lifestyle variables, and gender comparison.

	Total	Men	Women	*p*-Value ^(1)^
Adherence to the Mediterranean Diet				
(MEDAS score)				**<0.001**
(median (IQR)	7.0 (3.0)	7.0 (3.0)	8.0 (3.0)	
(mean ± SD)	7.4 ± 2.1	6.6 ± 2.2	7.7 ± 2.0	
MEDAS categories ^(2)^ (%/N)				**<0.001**
Low (≤5)	20.2/99	33.1/46	14.9/52	
Moderate (6–9)	62.7/307	56.1/78	65.3/228	
High (≥10)	17.1/84	10.8/15	19.8/69	
Lifestyle variables				
Meals per day (%/N)				**<0.001**
≤2	3.9/19	5.0/7	3.4/12	
3	21.0/103	33.6/47	16.0/56	
4	34.3/168	36.4/51	33.5/117	
5	31.0/152	16.4/23	36.7/128	
≥6	9.8/48	8.6/12	10.3/36	
Smoking (%/N)				**0.005**
Non-smoker	81.2/398	73.4/102	84.2/294	
Smoker	18.8/92	26.6/37	15.8/55	
Physical Activity (%/N)				0.811
Not regular	59.6/292	59.0/82	59.9/209	
Regular	40.4/198	41.0/57	40.1/140	
Time spent in Nature (%/N)				0.468
Never or occasionally	34.0/166	31.7/44	35.0/122	
Sometimes	35.9/176	36.7/51	35.5/124	
Frequently or almost all the time	30.1/148	31.7/44	29.5/103	
Sociability: time with family and friends (%/N)				0.78
Never or occasionally	7.3/36	10.8/15	6.0/21	
Sometimes	33.7/165	38.8/54	31.5/110	
Frequently or almost all the time	59.0/289	50.4/70	62.5/218	
Sleeping (%/N)				0.394
Less than six hours per night	14.7/72	17.3/24	13.8/48	
Six to seven hours per night	45.1/221	38.1/53	47.9/167	
Seven to eight hours per night	34.7/170	36.0/50	34.1/119	
Eight to ten hours per night	5.1/25	7.9/11	4.0/14	
More than ten hours per night	0.4/2	0.7/1	0.3/1	

MD = Mediterranean Diet. Sample size: total *N* = 490; Male, *N* = 139; Female, *N* = 349. ^(1)^ Mann–Whitney U tests were used to assess gender differences in ordinal and scale variables; chi square tests were used for nominal variables (significant differences in bold when *p*-values < 0.05); ^(2)^ 14-Mediterranean Diet Adherence Screener (MEDAS) adherence classes defined according to [28].

**Table 3:** Presence of diagnosed pathologies, BMI, and gender comparison.

	Total	Men	Women	*p*-Value ^(1)^
Pathologies (%/N)				0.27
Non diagnosed	74.7/349	78.2/104	73.3/244	
Diagnosed	25.3/118	21.8/29	26.7/89	
BMI (kg/m^2^) (median (IQR) /N)	23.4 (5.2)/472	24.3 (4.7)/135	22.6 (5.1)/335	<0.001
(mean ± SD)	24.2 ± 4.6	25.0 ± 3.7	23.9 ± 4.8	
BMI categories (%/N) ^(2)^				0.004
Underweight (<18.5)	3.0/14	0.7/1	3.9/13	
Normal (18.5; 24.9)	62.7/296	54.1/73	66.3/222	
Overweight (25; 29.9)	23.5/111	36.3/49	18.5/62	
Obese (≥30)	10.8/51	8.9/12	11.3/38	

Sample size is not constant due to missing data in different variables (pathologies, *N* = 467; BMI, *N* = 472) ^(1)^ Mann-Whitney U tests were used to assess gender differences in ordinal and scale variables; Chi square tests were used for nominal variables (differences were considered significant when *p*-values < 0.05); ^(2)^ BMI, Body Mass Index, calculated as weight (kg)/height^2^ (m^2^) and BMI categories were defined according to WHO [29].

**Table 4:** Participants’ subjective well-being (SWB) and gender comparison.

SWB Items (Scale 0 to 10) ^(1)^ (Median (IQR)/Mean ± sd) (Mean ± SD)	Total	Men	Women	*p*-Value
“Overall, to what extent do you feel that the things you do in your life are worthwhile?”	8 (2) 7.7 ± 1.6	8 (2) 7.6 ± 1.8	8 (2) 7.8 ± 1.5	0.256
“Overall, how satisfied are you with your life as a whole these days?”	7 (1) 7.3 ± 1.5	7 (1) 7.1 ± 1.6	7 (1) 7.3 ± 2.3	0.467
“How happy did you feel during the last week?”	7 (2) 6.9 ± 1.9	7 (2) 6.9 ± 2.1	7(2) 6.9 ± 1.9	0.948
“How worried did you feel during the last week?”	6 (4) 5.8 ± 2.5	6 (4) 5.7 ± 2.9	6 (4) 5.9 ± 2.5	0.545
“Did you feel depressed during the last week?”	3 (4) 3.3 ± 2.8	3 (6) 3.2 ± 2.9	3 (4) 3.3 ± 2.7	0.736
5-item SWB index ^(2)^	7 (2) 6.5 ± 1.5	7 (2) 6.5 ± 1.7	7 (2) 6.6 ± 1.5	0.892

^(1)^ 0 = not at all; 10 = completely/all the time; ^(2)^ The 5-item SWB index is the average of the items, with scales of worried and depressed inverted to have the same direction as the other variables before calculation of the 5-item index. *N*: total sample = 490, men = 139, women = 349.

**Table 5:** Profiles of subjective well-being (SWB) in the studied population.

SWB Profiles *N* (%)	Profile1 164 (31.4%)	Profile 2 275 (56.1%)	Profile 3 61 (12.4%)
Worthwhile life (Mean ± SD)	8.9 ± 0.9 ^a^	7.7 ± 1.0 ^b^	5.1 ± 1.7 ^c^
Overall life satisfaction (Mean ± SD)	8.4 ± 1.0 ^a^	7.2 ± 1.0 ^b^	4.7 ± 1.6 ^c^
Feeling of happiness (Mean ± SD)	8.5 ± 0.9 ^a^	6.7 ± 1.4 ^b^	3.8 ± 1.5 ^c^
Feeling of worry ^(1)^ (Mean ± SD)	6.0 ± 2.4 ^a^	3.5 ± 2.1 ^b^	2.6 ± 2.0 ^c^
Feeling of depression ^(1)^ (Mean ± SD)	9.3 ± 1.0 ^a^	6.1 ± 2.4 ^b^	3.4 ± 2.1^c^
5-item SWB index ^(2)^	8.2 ± 1.3 ^a^	6.2 ± 1.6 ^b^	3.9 ± 1.0 ^c^

^(1)^ Scales were inverted to have the same direction as the other variables: worthwhile life, overall life satisfaction and happiness, 0 = not at all, 10 = completely/all the time; worry and depression, 0 = all the time 10 = not at all; different letters in the same line indicate significant differences between profiles (*p* < 0.05); ^(2)^ The 5-item SWB index is the average of the items.

**Table 6:** Correlations of sociodemographic characteristics, BMI, SWB, and lifestyle habits with the adherence to the Mediterranean diet (MEDAS score).

Parameters	MEDAS Score (Spearman ρ/*p*-Values) ^(1)^
Sociodemographic	
Sex	**0.157/0.002**
Age	0.169/0.185
Time of residence in Portugal	0.031/0.546
Marital status	0.112/0.031
Household size	0.018/0.730
Education level	0.110/0.104
Employment status	**0.161/0.002**
Net income	0.154/0.003
BMI	−0.100/0.057
Lifestyle	
Smoking	−0.051/0.323
Sleeping hours per night	−0.013/0.810
Physical activity	−0.079/0.129
Time spent in Nature	**0.182/<0.001**
Sleeping	−0.062/0.235
Time spent with family or friends	−0.030/0.568
Meals per day	**0.243/<0.001**
SWB	
Worthwhile life	0.127/0.012
Overall life satisfaction	0.098/0.053
Feeling of happiness	0.019/0.704
Feeling of worry	−0.109/0.032
Feeling of depression	−0.070/0.169

^(1)^ All correlations controlled for potential confounders; significant values in bold, considered when *p* < 0.0025 (adjusted by Bonferroni’s Correction). BMI, Body Mass Index; SWB, Subjective well-being.

## Supplementary Materials

The following are available online at https://www.mdpi.com/2072-6643/12/12/3837/s1, S1: Questionnaire MeDiWeB: Assessment of the impact of Mediterranean diet and other lifestyle factors on well-being.
