# Supplementary material for: Mediterranean Diet Adherence and Subjective Well-Being in a Sample of Portuguese Adults
Source: Nutrients. 2020 Dec 16;12(12):3837. doi: 10.3390/nu12123837 (PMC7765516; doi:10.3390/nu12123837)
Supplement: Supplementary file 1 [file nutrients-12-03837-s001.pdf]

Questionnaire MeDiWeB: Assessment of the impact of Mediterranean diet and other lifestyle factors on well-being

THIS IS A CONSORTIUM AGREEMENT BETWEEN THE FOLLOWING COUNTRIES OF THE MEDITERRANEAN AREA: PORTUGAL, SPAIN, GREECE, BULGARIA, REP. OF NORTH OF MACEDONIA, ITALY AND CYPRUS

PRINCIPAL INVESTIGATOR/S: DR. Paula Pinto

RESEARCH CENTRE: POLYTECHNIC INSTITUTE OF SANTAREM (PORTUGAL)

SPECIFIC INFORMATION FOR EACH COUNTRY

RESEARCHER RESPONSIBLE (COUNTRY): DR. ... (...)

POSITION: ...

RESEARCH CENTRE (COUNTRY): ...

LOCATION WHERE THE STUDY IS CONDUCTED (COUNTRY): ... (...)

Contact telephone:

email address:

INFORMATION SHEET FOR THE PARTICIPANT

We kindly ask you to read with attention the information that follows before you decide to proceed with the questionnaire. In this section, we would like to inform you about this study that has been approved by the Ethics Committee of ... (each country) and in which we invite you to participate as a VOLUNTEER.

GENERAL DESCRIPTION OF THE STUDY

The MeDiWeb study is part of an international collaborative project between Universities and Research Centres from several Mediterranean countries led by Portugal and in which Spain, Greece, Bulgaria, Republic of Macedonia, Italy and Cyprus also participate. The main objective of this project is to find out the relationship and impact of our lifestyle and food habits with our daily energy, level of stress, emotional state and personal satisfaction by means of a specific questionnaire designed for this purpose. For this, it is essential that we have your participation and sincere responses.

NATURE OF THE PARTICIPATION

First of all, and very important, we would like to assure you that your participation in this survey will be completely CONFIDENTIAL. This questionnaire has been sent to you via email with a direct link to the survey. The specific configuration of the system has been selected to eliminate the possibility of tracking down your email contact. This way we will make sure that all your data will be used in total ANONYMITY.

Your participation in this study is also completely VOLUNTARY and thus, you can decide whether to take part or not in the survey. You may also stop and quit the survey at any time during your participation by getting out of the questionnaire and simply not sending it. If you proceed till the end, once you send the filled questionnaire we shall receive the data in an ANONYMOUS way and, these data will be analysed using appropriate statistical tools together

with all the data collected from all the participants. The whole set of data will be used for the preparation of scientific publications.

The survey will take you only a FEW MINUTES to complete.

BENEFITS

The main initial benefits of this study will be of scientific nature and the results of the survey are expected to provide improved knowledge and understanding of the association between our lifestyle and food habits and our perception of well-being, in particular, of whether we feel stressed, fatigued or energetic in our daily life and what is, in general, the level of satisfaction with our life. The direct benefits will be one (or several) communications of these results in the form of scientific articles and (or) presentations at specific Conferences in the area of Mediterranean lifestyle and well-being.

On a second step, we envisage the possibility of being able to prepare some general recommendations as to how to implement some behavioural changes that may contribute to improve our lifestyle and well-being. These recommendations may be disseminated through normal communication media (journals, website, radio, consumers associations, etc).

RISKS

Since this is a completely ANONYMOUS questionnaire and the protocol does not include any interaction with the volunteers nor the taking of any (bio)samples, we cannot envisage any risk for the participants. We shall not contact with the participants at any time and we will not request his/her presence. The only intervention from the side of the participants is to dedicate a few minutes to answer the questionnaire and to send it.

RESPONSIBILITY OF THE RESEARCHERS TEAM

In this particular study, there is no interaction between the researchers and the participants neither the need to take any biological samples, genetic testing or health risk for the participants. There is only one item in which we ask the participant to let us know whether he/she has been diagnosed with any particular disease. Once again, since this is a fully CONFIDENTIAL participation, this information will be used for statistical analyses in the context of the whole population taking part in the study. We shall not request any further information about health or the health of relatives. Therefore there is no need for sanitary assistance or insurance policy.

The participation in this study is completely VOLUNTARY and it does not have associated any stipend.

As the researchers in charge of this study, we are fully responsible for assuring that the protocol indicated will be the one to follow throughout the duration of the whole study.

PROCEDURE

As already stated, the protocol for participation in this study is very simple. You will receive an email with a link to the questionnaire implemented via the Google survey tool. Once you open it, you will first read the information in this Section 1 and if you agree on taking part in the study you will need to accept the participation by signing the consent form included further below. By clicking in the declarations specified in this consent form and by clicking in the corresponding box giving your consent and then choosing to go to the next section, we shall

consider that you have read and understood the study and the protocol and that you give your consent to participate in it.

Nevertheless and as also stated before you may quit your participation in the study by getting out of the questionnaire at any time and not sending it.

INFORMATION RELATIVE TO DATA

The data collected during this survey will be saved into specific Excel files designed for it. The data from all the participants will be ANONYMOUSLY added to the file and saved in the computer designated by the scientist(s) implicated within each of the participant Universities and Research Institutions. Since one of the objectives may be to compare individuals from different countries, the data may be shared at one point between all the members of the consortium for global statistical analyses. Once the data have been analysed and published, we shall proceed with their destruction by erasing the files. The time gap between data collection and destruction will depend on the analyses to be carried out and the finalisation of the publications and data full exploitation.

FOR MORE INFORMATION

As a potential participant you have the right to obtain any clarification about potential doubts that you may have at any time. You can ask for more detailed information about this investigation. For this you can contact directly with the researcher responsible of the study in your country whose contact details are indicated at the beginning of this document. If you consider that all your doubts and concerns have been conveniently solved and you are convinced to take part in the study, please, proceed by signing the informed consent form exposed below.

In the name of the MeDiWeB team, we thank you very much for your enthusiasm and support.

Paula Pinto

(Prof Coordenadora, Instituto Politécnico de Santarém, Escola Superior Agrária)

INFORMED CONSENT FORM I hereby DECLARE that:

I have read the Information Sheet for the participant and attained sufficient information about the project as well as about the main objectives of it. I have also understood the conditions of my participation in this study: 1) Data CONFIDENTIALITY; 2) VOLUNTARY participation (I can freely stop answering the responses to the questionnaire at any time point and quit the survey without any consequences for me); 3) My data will only be used ANONYMOUSLY for statistical analyses and scientific publication(s).

I confirm that I GIVE my consent to take part in this study and that it is my intention to continue with the questionnaire/ I DO NOT GIVE my consent to take part in the proposed study

Lifestyle and Well-being: In this section the first set of questions are general population demographic measures; then we ask you to reflect on some aspects of your life, related to well-being. The answers are confidential and individual. Answers will not be public under any circumstance.

2.1. Please, indicate your sex
2.2. Please, indicate your age (in years)

2.3. Please, indicate your nationality
2.4. Please, indicate your education level
Middle school/ High School/ University/ Master/ PhD
2.5. Please, indicate your marital status
Single/ Married or in an analogous relationship of affect/ Divorced or separated/ Widowed
2.6. Please, indicate the total number of persons living in your household (Including yourself)
2.7. Please, indicate the country of your current permanent residence
2.8. How many years have you been living in the country of your current permanent residence?
Questions 9 to 13 were adapted from OECD guidelines on measuring subjective well-being (OECD
2013)
2.9. Overall, how satisfied are you with your life as a whole these days? This question asks how satisfied you feel, on a scale from 0 to 10. Zero means you feel "not at all satisfied" and ten means you feel "completely satisfied".
2.10. Overall, to what extent do you feel that the things you do in your life are worthwhile? This question asks how you feel, on a scale from 0 to 10. Zero means "Not at all worthwhile" and ten means "completely worthwhile".
2.11. How happy did you feel during the last week? This question asks how you feel, on a scale from 0 to 10. Zero means you feel "not at all happy" and ten means you feel "happy all the time".
2.12. How worried did you feel during the last week? This question asks how you feel, on a scale from 0 to 10. Zero means you feel "not at all worried" and ten means you feel "worried all the time".
2.13. Did you feel depressed during the last week? This question asks how you feel, on a scale from 0 to 10. Zero means you feel "not at all depressed" and ten means you feel "depressed all the time".
Questions 14 to 16 assess the energy level and were adapted from the Lee's fatigue scale (Lee, K.;
Hicks, G.; Nino-Murcia, G. Validity and Reliability of a Scale to Assess Fatigue. Psychiatry Research,
1990, 36:291-298)
2.14. Last week, how energetic did you normally feel in the middle of the day? This question asks how you feel, on a scale from 0 to 10. Zero means you feel "not at all energetic" and ten means you feel "extremely energetic".
2.15. Last week, how efficient did you normally feel in the middle of the day? This question asks how you feel, on a scale from 0 to 10. Zero means you feel "not at all efficient" and ten means you feel "extremely efficient". Consider efficient as focused and well organized.
2.16. Last week, how tired did you normally feel in the middle of the day? This question asks how you feel, on a scale from 0 to 10. Zero means you feel "not at all tired" and ten means you feel "extremely tired".
2.17. During last week, how often did you feel nervous and stressed? This question asks how you feel, on a scale from 0 to 10. Zero means you feel "never" and ten means you feel "stressed all the time".
2.18. During last week, how often did you feel that you were unable to cope with all the things you had to do? This question asks how you feel, on a scale from 0 to 10. Zero means you feel "never" and ten means you feel "all the time".
2.19. During last week, how often did you feel confident about your ability to handle your personal problems? This question asks how you feel, on a scale from 0 to 10. Zero means you feel "never" and ten means you feel "all the time".

2.20. Please, indicate your employment status during the last year
Student/ Employed/ Seeking for my first job/ Unemployed part of the year/ Unemployed/ Pensionist (retired, disability)/ Housework
2.21. If you are employed, how would you classify the level of satisfaction with your job?
This question asks how satisfied you feel, on a scale from 0 to 10. Zero means you feel "not at all satisfied" and ten means you feel "completely satisfied".
2.22. Please, indicate your profession
2.23. Please, indicate your weight in kilograms
2.24. Please, indicate your height in centimetres
2.25. Do you currently suffer from any diagnosed pathology? Please answer yes or no; if yes, please specify which pathology
2.26. Are you currently a smoker?
Yes/ No
2.27. What type of activity do you have daily (at work, home)
I am normally sat down and I don´t walk very much (administrative work, taxi or bus driver)/ I spend some time walking but I don´t do any strenuous effort (salesperson, industry)/ I spend quite a lot of time walking and I do frequent strenuous efforts (postman, cleaner, hauler)/ I do a lot of strenuous efforts, hard work and activity (builder, dockworker, farmer).
2.28. What type of activity do you practice more habitually at leisure?
Activities that do not require physical activity (reading, watching TV)/ Relaxing activities (walking, gardening, slow biking) sometimes per week/ Practise sport and (or) strong physical activity.
2.29. If you practise sport, on average, last month, how frequently did you practise exercise? (fast walking, running, cycling, swimming, gymnastics, other sports)
Never/ Occasionally, but not regularly/ Regularly, less than 150 minutes per week/ Regularly, 150 minutes or more per week
2.30. On average, last month, how many hours did you sleep per night?
Less than six hours per night/ Six to seven hours per night/ Seven to eight hours per night/ Eight to ten hours per night/ More than ten hours per night
2.31. Do you take a short sleep ('siesta') during the day?
No/ Yes, but only occasionally/ Yes, frequently
2.32. If you do sleep 'siesta', please, indicate the duration of it
Less than 30 minutes/ Between 30 minutes and 1 hour/ More than 1 hour
2.33. During the last month, how often did you spend time ...
... in contact with nature?
... with your family?
... with friends?
Never/ Occasionally/ Sometimes/ Frequently/ Almost all the time
2.34. Who do you normally have the main meals of the day with? (lunch, dinner)
Alone/ In the company of colleagues, friends or family.
2.35. How would you classify your satisfaction with your sexual life? This question asks how satisfied you feel, on a scale from 0 to 10. Zero means you feel "not at all satisfied" and ten means you feel "completely satisfied".
2.36. What is, approximately, your household's monthly net income? (in Euros)

Food Habits: This section asks you about foods and frequency of consumption. Please answer the following questions thinking about your habits in the last month.

3.1. How many meals do you have daily?
(including, for example, breakfast, lunch, dinner, morning and afternoon snacks, and supper)
Two or less/ Three/ Four/ Five/ Six or more

3.2. Do you use olive oil as your main culinary fat?
Yes/ No
3.3. How many tablespoons of olive oil do you consume per day?
(Including olive oil used for cooking, salads, out of house meals, etc)
One or less/ Two or three/ Four or more
3.4. How many vegetable servings do you consume per day?
(Including cooked and raw vegetables; potatoes and beans are not included). One serving = one large cup or half a large plate)
Less than one/ One/ Two/ Three or more
3.5. How many servings of fresh fruit do you consume per day?
One serving = one fruit unit of medium size, one large cup of sliced fruit, one slice of melon or watermelon of medium size, or one cup of freshly squeezed juice.
Less than one/ One/ Two/ Three or more
3.6. How many servings of red meat (pork, veal/beef, lamb), or red meat products (hamburgers, sausages, ham) do you consume per week?
One serving = 100 to 150 g = a quarter to half a meal dish
One or less/ Two to four/ Five to six/ Seven or more
3.7. How many servings of butter, margarine, or cream do you consume per day?
One serving = 12 g = one dessert spoon of butter and margarine; 2 table spoons for cream
Less than one/ One/ More than one
3.8. How many sweet beverages do you consume per day?
Less than one/ One/ More than one
3.9. How many glasses of wine do you consume per week?
One or less (occasionally)/ Two to six (sometimes but not daily)/ Seven to fourteen (one or two glasses per day)/ More than fourteen (more than two glasses per day)
3.10. How many servings of legumes do you consume per week? (beans, peas, chickpeas, lentils) One serving = 150 g = 1 plate or 1 cup
Less than one/ One/ Two/ Three or more
3.11. How many servings of fish or shellfish do you consume per week?
One serving = 100 to 150 g = a quarter to half a meal dish
Less than one/ One/ Two/ Three or more
3.12. How many times per week do you consume commercial sweets or pastries (not homemade), such as cakes, cookies, biscuits, or custard?
Less than one/ One/ Two/ Three/ Four or more
3.13. How many servings of non-salted nuts do you consume per week?
One serving = 30 g = one handfull (include non salted peanuts, almonds, hazelnuts, chestnut, walnuts)
Less than one/ One/ Two/ Three or more
3.14. Do you preferentially consume chicken, turkey or rabbit meat, or a vegetarian protein source, instead of red meat (veal/beef, pork, lamb) or any derived products (hamburgers, sausages, ham)?
Yes/ No
3.15. How many times per week do you consume dishes cooked with tomato or tomato sauce, onion and (or) garlic, and olive oil?
Less than one/ One/ Two or more
3.16. How many servings of milk, yogurt or cheese do consume per day?
One serving = 1 cup of milk, or 1 yogurt, or 2 thin slices of cheese
Less than one/ One/ Two/ Three or more
3.17. Do you eat preferentially low fat milk, yogurt, or cheese?
Yes/ No
3.18. Do you eat preferentially whole cereals?

Whole cereals include non refined wheat products such as whole bread and pasta, rye bread, and
brown rice. Refined cereals include white wheat bread, white pasta, and white rice).
Yes/ No
3.19. How many cups of caffeinated drinks do you have per day?
(Including coffee, black or green tea, cola drinks, or other energy drinks)
Less than one/ One to three/ More than three
3.20. How many cups of infusions/herbal teas do you have per day?
Less than one/ One to three/ More than three
3.21. How much water do you drink per day?
Less than one liter (4 glasses)/ One to two liters (4 to 8 glasses)/ More than two liters (more than 8 glasses)
